# Capped ZnO quantum dots with a tunable photoluminescence for acetone detection

**DOI:** 10.1039/d3ra00491k

**Published:** 2023-06-01

**Authors:** Goerget Saber, Ali El-Dissouky, Gamal Badie, Shaker Ebrahim, Azza Shokry

**Affiliations:** a Department of Chemistry and Physics, Faculty of Education, Alexandria University El-Shatby 21526 Alexandria Egypt goergetsaber@alexu.edu.eg; b Department of Chemistry, Faculty of Science, Alexandria University Ibrahimia, P. O. Box 426 Alexandria Egypt; c Department of Materials Science, Institute of Graduate Studies and Research (IGSR), Alexandria University 163 Horrya Avenue, El-Shatby, P. O. Box 832 Alexandria Egypt

## Abstract

Acetone is a dangerous material that poses a major risk to human health. To protect against its harmful impacts, a fluorescent biosensor 3-aminopropyl triethoxysilane capped ZnO quantum dots (APTES/ZnO QDs) was investigated to detect low concentrations of acetone. Numerous techniques, including Fourier transform infrared (FTIR), energy dispersive X-ray (EDX), X-ray photoelectron spectroscopy (XPS), X-ray diffraction (XRD), high-resolution transmission electron microscopy (HRTEM), zeta potential, UV-vis absorption, and photoluminescence (PL), are used to thoroughly verify the successful synthesis of pristine ZnO QDs and APTES/ZnO QDs. The HRTEM micrograph showed that the average size distributions of ZnO QDs and APTES/ZnO QDs were spherical forms of 2.6 and 1.2 nm, respectively. This fluorescent probe dramatically increased its sensitivity toward acetone with a wide linear response range of 0.1–18 mM and a correlation coefficient (*R*^2^) of 0.9987. The detection limit of this sensing system for acetone is as low as 42 μM. The superior selectivity of acetone across numerous interfering bioanalytics is confirmed. Reproducibility and repeatability experiments presented relative standard deviations (RSD) of 2.2% and 2.4%, respectively. Finally, this developed sensor was applied successfully for detecting acetone in a diabetic patient's urine samples with a recovery percentage ranging from 97 to 102.7%.

## Abstract 2

Preparation of APTES/ZnO QDs and the PL enhancement of APTES/ZnO QDs toward acetone concentrations (0.1–18 mM).

## Graphical abstract

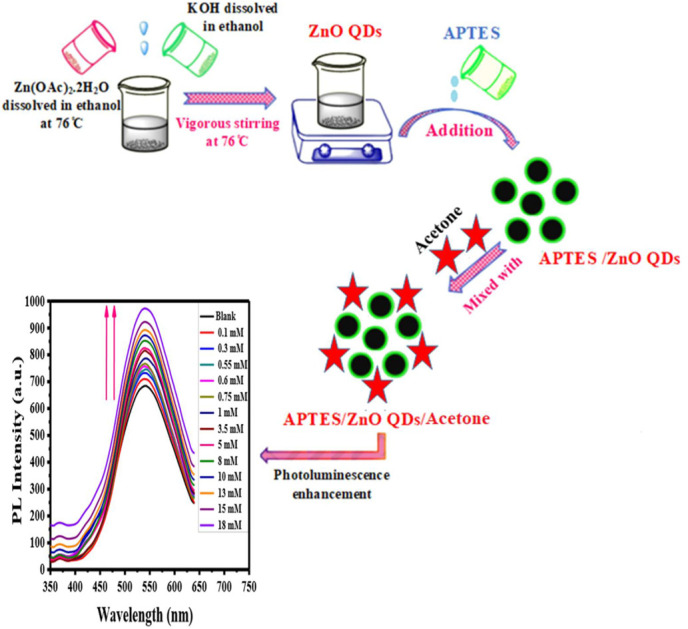

## 1 Introduction

Quantum dots (QDs) are nanocrystals with distinctive electrical and optical characteristics in a size regime below 10 nm.^1,2^ QDs are potent fluorophores frequently utilized as sensors for various applications, including medical,^3^ biological,^4^ and environmental.^5^ The surface capping of QDs is the key for improving the photoluminescence efficiency, preventing QDs from aggregating, creating a shielding barrier that protects and stabilizes the QDs surface.^6,7^ ZnO QDs are a promising candidate for biological applications and are used as an alternative to other semiconductors.^8,9^ They are used in commercial sunscreens and cosmetics to protect skin against UV radiation.^10^ There are diverse approaches for synthesizing different-sized ZnO QDs, such as chemical precipitation, combustion and hydro/solvothermal methods.^11^ The sol–gel route is the most successful approach for preparing quantum-sized ZnO. This process is inexpensive, more reliable, affordable, owns perfect reproducibility, and it allows controlling of the particle's size and form.^12,13^

Acetone (CH_3_COCH_3_) is a harmful organic solvent, a primary biomarker in diabetes mellitus, and one of the chronic diseases with the fastest growth rate.^14^ It can be found in human blood, urine, and breathed air as a byproduct of regular metabolism.^15^ It is produced through the ketosis process, at which the body's supply of carbohydrates runs out, and there is a lack of insulin in the pancreas, resulting in the breakdown of fats in the cell of the body better than interacting with glucose.^16^ According to World Health Organization (WHO), the permissible limit to exposure of acetone gas was set lower than 173 mg L^−1^ (∼2.9 mM) and prolonged inhalation to higher this acetone concentration can lead to kidney damage, headache, eye and respiratory diseases.^17^ Several techniques have been used to detect acetone concentration, such as FTIR spectroscopy, chromatography, and chemical method, but these methods are expensive and complex.^15,18,19^ Therefore, the need for a less risky, low-costly, and high-performance method, which may sense the low concentration of CH_3_COCH_3_ levels (ppb), is urgent to diagnose people with diabetics early in humans.^14^ Metal oxide semiconductors have been evaluated as candidates amongst various active sensing matters utilized for detecting acetone because of their remarkably high sensitivity, ultrahigh porosity, and extraordinarily high surface area.^20,21^

As a consequence, it is urgent to develop effective technique that can target this goal. APTES is used as an effective new capping agent in synthesis quantum dots to improve sensitivity and optical properties besides the most stabilization for QDs and also detecting acetone more than that reported in literature.^15,18,19^

This work uses the sol–gel method to prepare pristine ZnO QDs and APTES/ZnO QDs. Analytical techniques such as FT-IR, XPS, EDX, XRD, HRTEM, zeta potential, UV-visible, and PL spectra are used to discuss the morphological, structural, surface properties and optical characteristics of these QDs. The linearity, sensitivity, dynamic range, and detection limit of acetone ranging from 0.1 to 18.0 mM are all determined using the PL property of these QDs. The pH influence in the range of 2–10 on the PL efficiency and intensity of APTES/ZnO QDs is studied. The selectivity and interference of APTES/ZnO QDs in the existence of several interfering bioanalytics are investigated. The possible mechanism for the interaction between APTES/ZnO QDs and acetone is proposed and discussed. Also, the repeatability and reproducibility experiments is examined. For the first time, as far as we know, APTES/ZnO QDs are utilized as a photoluminescent enhancement biosensor for the quantification of acetone.

## 2 Experimental

### 2.1. Chemicals and reagents

Zinc acetate dihydrate (Zn(OAc)_2_·2H_2_O, 98.5%) was supplied by Oxford Instruments, India. Potassium hydroxide (KOH, 85%) was purchased from El-Gomhouria Chem-Co., Egypt. Acetone (98.5%) was obtained from Adwic, Egypt. Ethanol (99.9%) was supplied by International Co. for supply and Medical Industries, Darmstadt, Germany. APTES (99%), uric acid (95.5%), hydrochloric acid (HCl, 36.0%), calcium carbonate (99.5%) and glucose (99%) were obtained from Sigma-Aldrich, UK. Phosphate buffer saline (PBS, 99.9%) (pH 7) was obtained from Chem-Lab, USA. Sodium hydroxide (NaOH, 98.5%) was supplied by El-Nasr Co., Egypt. Cholesterol (99.9%) was received from Vitroscent, UK. Diabetic urine samples are obtained from a clinical laboratory.

### 2.2. Preparation of pristine ZnO QDs

1.0 mmol of Zn(OAc)_2_·2H_2_O solution was prepared by dissolving 0.22 g of Zn(OAc)_2_·2H_2_O in 100 mL ethanol with stirring for 30 min at 76 °C and left to cool to 33 °C. KOH (3.0 mmol) solution was prepared by dissolving 0.17 g of KOH in 8 mL ethanol. The obtained KOH solution was dropped wisely and added to the Zn(OAc)_2_·2H_2_O solution under vigorous stirring at 76 °C for 30 min until a white colloidal solution of ZnO QDs was obtained. Then, 10 mL of absolute ethanol was added to the colloidal solution under vigorous stirring at room temperature for another 30 min.

### 2.3. Preparation of APTES capped ZnO QDs

APTES/ZnO QDs were prepared by adding 0.05 M APTES in ethanol solution (10 mL) to the as-prepared ZnO QDs solution under vigorous stirring at room temperature for 30 min. The as-prepared capped and uncapped ZnO QDs were separated by centrifuging process (focus serial no.: 1107, Spain) at 6500 rpm for 10 min. The QDs were washed five times with absolute ethanol to remove unreacted molecules. Finally, the APTES/ZnO QDs and ZnO QDs were dried in an oven at 60 °C for 2 h.

### 2.4. Characterization of ZnO QDs and APTES/ZnO QDs

The absorption spectra of the prepared pristine ZnO QDs and APTES/ZnO QDs dispersed in ethanol were recorded using a UV-visible spectrophotometer (Evolution 300, Thermo Scientific, USA). The PL spectra of these QDs were examined to investigate the emission characteristics. A fluorescence spectrophotometer performed PL measurements (PerkinElmer LS-55). At room temperature, the measurements were made. Both emission and excitation slits were set at 10.0 nm. The crystallinity of ZnO QDs and APTES/ZnO QDs were verified by XRD (Bruker-AXS D8 Discover) with copper Kα radiation (*λ* = 1.54060 Å) and operates on 40 kV voltage and 20 mA current. The functional groups in these QDs were evaluated using FT-IR analysis (Spectrum BX 11-LX 18-5255 PerkinElmer) with KBr pellets in the wavenumber range of 400–4000 cm^−1^. The energy dispersive X-ray technique (EDX) connected to a SEM instrument was used to study the elemental analysis (JSM-IT 200). The chemical composition and changes in the chemical binding states of ZnO QDs and APTES/ZnO QDs were examined utilizing XPS spectra (Themo Fisher Scientific, USA) with a monochromatic X-ray ALK-alpha radiation spot size 400 micro at pressure 9–10 mbar with full spectrum energy 50–200 eV. The particle size, morphology and selected area electron diffraction (SAED) of the as-prepared QDs were studied using HRTEM (JEOL, JEM-2100 LaB6). The surface charges of samples were estimated using *ξ*-potential measurement (Zetasizer Malvern Nano-ZS).

### 2.5. Photoluminescence quantification of acetone

A 0.15 mL of acetone was dissolved in 100 mL ethanol to prepare a stock solution (20 mM) of acetone. A fixed volume of APTES/ZnO QDs in ethanol solution was used for sensing acetone as follows. At room temperature, 0.1 mL of capped QDs solution reacted with 0.1 mL of various acetone concentrations from 0.1 to 18.0 mM prepared from appropriate dilution of stock solution. The mixture was diluted to 10.0 mL with absolute ethanol in a certified measuring flask, shaken thoroughly by vortex for 10 min, and then incubated for 25 min at room temperature. The PL measurements were performed at an *λ*_ex_ of 330 nm and collected at an *λ*_em_ of 540 nm, and a standard calibration curve is sketched from these results.

For analytical parameters optimization, the following eqn (1) is carried out:^22^

1 Δ*F* = *aC*_acetone_ + *b*

where Δ*F* is the PL enhancing intensity, *C*_acetone_ signifies the acetone concentration, and *a*, and *b* are the calibration curve's slope and intercept, respectively.

The PL enhancing intensity (Δ*F*) was used based on eqn (2):^22^

2 Δ*F* = *F* − *F*_0_

where *F* and *F*_0_ are the PL intensities of APTES/ZnO QDs in acetone presence and absence, respectively.

The limit of detection (LOD) value of APTES/ZnO QDs was obtained using eqn (3):^16^3
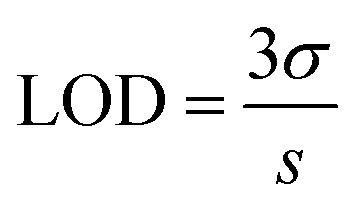
where *σ* is the blank sample's standard deviation and *s* is the slope between the PL intensity and acetone concentration.

To improve the experimental detection circumstances, the impact of numerous parameters, such as pH and the response time between APTES/ZnO QDs and acetone, was studied to enhance the experimental detection conditions.

The effect of pH was investigated by preparing different pHs solutions (2 to 10) from PBS (pH = 7), and the pH of these solutions was adjusted utilizing 1.0 M HCl and 0.1 M NaOH. For PL measurements, 0.1 mL of APTES/ZnO QDs is added into a measuring flask (10 mL) and completed to the mark with different pHs.

In addition, the selectivity and interference of APTES/ZnO QDs toward acetone were examined by measuring the PL intensity in the presence of the potential interference bioanalytics in human urine, such as glucose, cholesterol, calcium carbonate, uric acid and a mixture of them (15.0 mM). The standard deviations were used to calculate the error bars. All measurements were tested and repeated three times by adding 0.1 mL of the luminescent probe and 0.1 mL of interfering substances to 9.8 mL of ethanol after being shaken thoroughly by vortex for 10 min and incubated at room temperature for 25 min.

### 2.6. Acetone detection in actual human urine samples

The as-luminescent APTES/ZnO QDs probe was applied for sensing acetone in three urine samples obtained from diabetes patients from a local clinical analysis lab. The urine specimen was collected in a tube without preservatives and pretreatment for PL measuring. A standard method for detecting acetone concentration in the urine sample was used by immersing a test strip (Keto-Diabur-Test 5000, Roche) obtained from this local clinical lab. Using the reference range supplied with the strips for urinalysis acetone laboratory analysis, at which (+) 5 mM is referred to as slightly raised, (++) 10 mM is referred to as raised, and (+++) 15 mM is referred to as highly increased concentration. The readings were checked after 2 minutes following the manufacturer's instructions.^23^ For the PL response of the urine specimen, 0.1 mL aliquot of this sample is added to 0.1 mL of the probe and 9.8 mL of absolute ethanol. For 10 minutes, thoroughly mixed with a vortex, and then incubated for 25 minutes at room temperature. All patients provided written informed consent.

## 3 Results and discussion

### 3.1. Structural properties of ZnO QDs and APTES/ZnO QDs

**Fig. 1:**
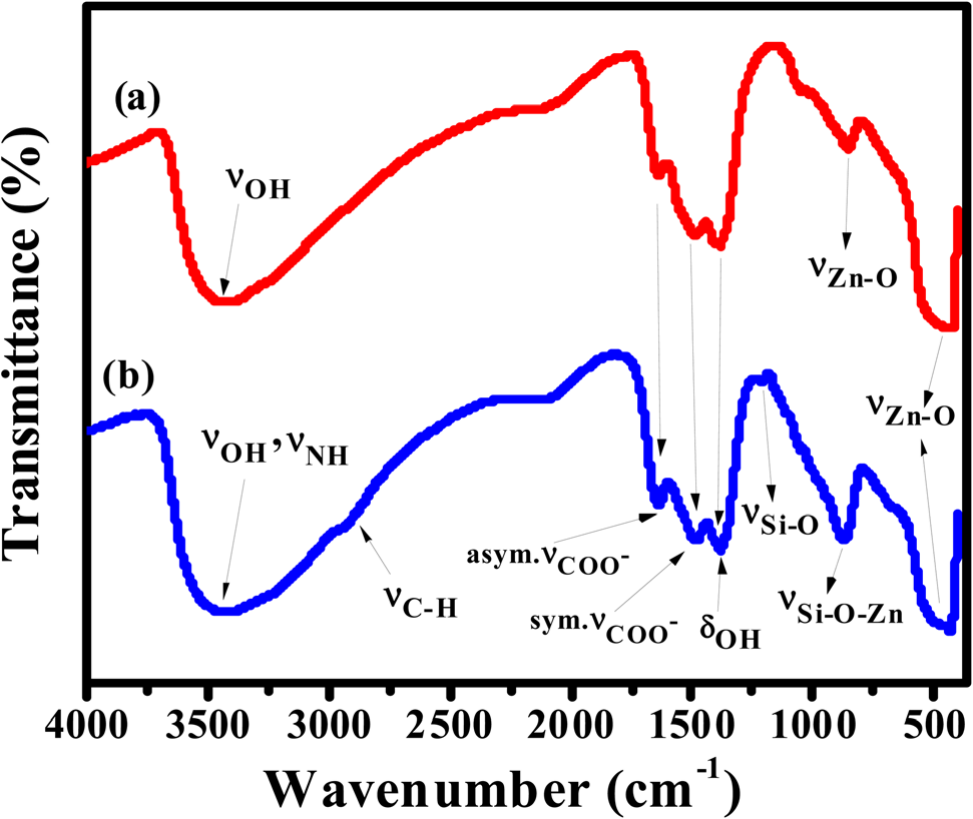
FTIR spectra of (a) ZnO QDs, and (b) APTES/ZnO QDs.

APTES/ZnO QDs and uncapped ZnO QDs are characterized by FTIR, EDX and XPS spectra. The FTIR spectra of ZnO QDs and APTES/ZnO QDs are displayed in Fig. 1. These spectra display a broad band at 475 and 462 cm^−1^ of *ν*_Zn–O_.^24,25^ The red shift of *ν*_Zn–O_ in the case of APTES/ZnO QDs relative to ZnO QDs is referred to the higher effective mass of Zn–O upon capping with APTES.^13^ The spectra exhibit weak bands at 1410 and 1670 cm^−1^ in both species which could be referred to the symmetric and asymmetric vibration of the acetate group *ν*_COO_^−^ bonded to zinc ion.^10,26^ The spectrum of ZnO QDs, Fig. 1(a), has a broad medium band at 3425 cm^−1^ and a weak one at 1370 cm^−1^ attributable to *ν*_OH_ and *δ*_OH_, respectively.^27,28^ The strong absorption peak observed at 880 cm^−1^ is assigned to *ν*_Zn–O_.^29^ The APTES/ZnO QDs spectrum, Fig. 1(b), reveals new bands at 2930, 1113 and 870 cm^−1^ corresponding to *ν*_C–H_ of the –CH_2_– and –CH_2_CH_3_–, *ν*_Si–O_ and *ν*_Si–O–Zn_, respectively.^10,26,27^ The appearance of these new bands indicates the success of the formation of APTES/ZnO QDs. Furthermore, the most distinguishing band at 3300–3500 cm^−1^ is because of *ν*_N–H_ of the APTES overlapped with *ν*_O–H_^30^ and has become more border in the uncapped ZnO QDs (Fig. 1(b)).

**Fig. 2:**
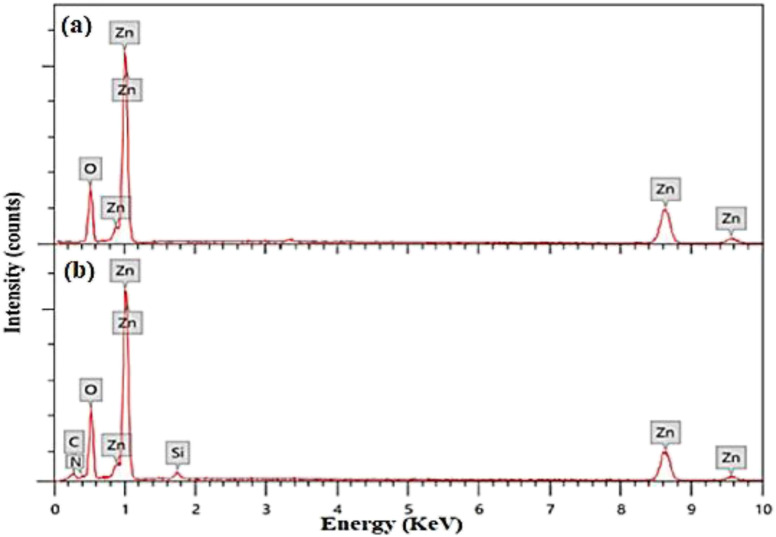
(a and b) EDX spectra of ZnO QDs and APTES/ZnO QDs, respectively.

The elemental analysis of capped and pristine ZnO QDs are determined *via* the EDX analysis and clarified in Fig. 2(a) and (b). The EDX spectrum of APTES/ZnO QDs, Fig. 2(b), illustrates peaks corresponding to C (8.98%), N (0.98%), Si (2.11%), O (32.80) and Zn (55.13%). However, peaks characteristic of only Zn (74.37%) and O (25.63%) appeared in the case of ZnO QDs, Fig. 2(a). The EDX data agree with the FTIR data, which support the formation of ZnO QDs and APTES/ZnO QDs.

**Fig. 3:**
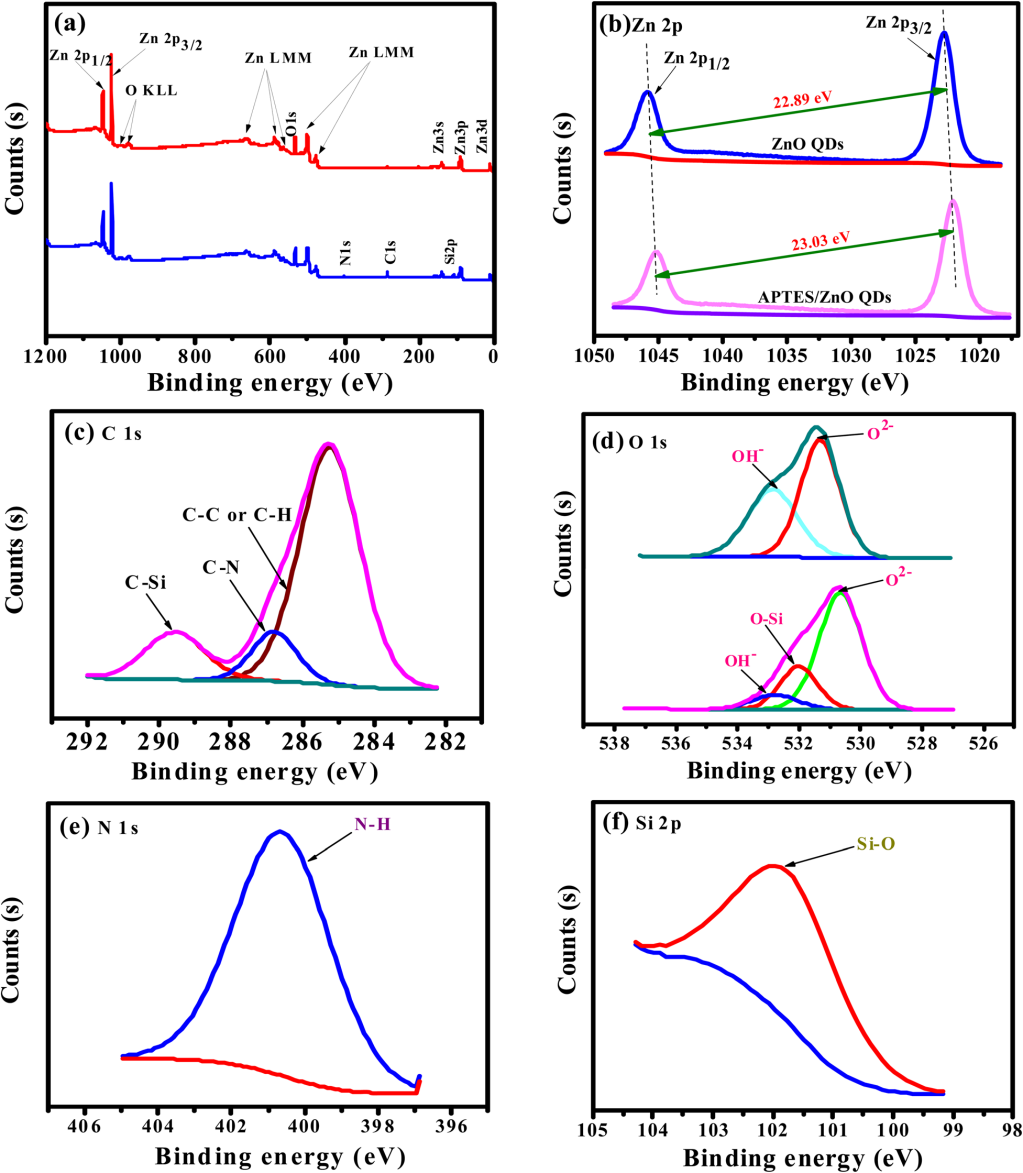
XPS full-range images of (a) ZnO QDs (red line) and APTES/ZnO QDs (blue line); deconvolution (b), (c), (d), (e) and (f) the core levels of Zn (2p), C (1s), O (1s), N (1s) and Si (2p), respectively.

Formation and changes in binding states of ZnO QDs and APTES/ZnO QDs were also confirmed *via* XPS analysis and shown in Fig. 3(a)–(f). The full scan spectra of both species, Fig. 3(a), exhibit only signals characteristic of Zn (2p) and O (1s) in the case of ZnO QDs. On the other hand, APTES/ZnO QDs spectrum shows peaks characteristic of Zn (2p), O (1s), C (1s), N (1s) and Si (2p). Both spectra display the Auger peaks characteristic of Zn LMM and O KLL.^31^ The atomic ratios of the surface of ZnO QDs are assessed to be 52.86% and 47.14% for Zn and O, respectively. The atomic percentages of the chemical compositions for APTES/ZnO QDs are 1.99, 11.08, 4.43, 43.16 and 48.35%, corresponding to N, C, Si, Zn and O, respectively. The XPS spectral patterns and atomic ratios confirm the success of the preparation of both species of QDs. The deconvolution of the Zn (2p) peaks, Fig. 3(b), in the case of ZnO QDs, displays two distinct peaks of comparable intensities at 1022.80 and 1045.69 eV due to the spin–orbit coupling of Zn 2p_3/2_ and Zn 2p_1/2_ levels, respectively. Both components' peak positions and the binding energy separation of 22.89 eV strongly confirm the oxidation state of 2+ of Zn^2+^ in the ZnO QDs.^32–34^ The deconvolution of zinc peak in the spectrum of APTES/ZnO QDs, Fig. 3(b), exhibits Zn 2p_3/2_ and 2p_1/2_ core levels at 1021.93 and 1044.96 eV, respectively. The binding energy difference of 23.03 eV and the relative intensity of the two components confirm that the oxidation state 2+ of zinc ions remains unchanged in the presence of APTES.

Peak fitting of the narrow scans for C (1s), O (1s), N (1s), and Si (2p) allows identifying several chemical bonds that are consistent with the presence of APTES as presented in Fig. 3(c)–(f). Fig. 3(c) demonstrates the C 1s with three peaks at 285.25, 286.8 and 289.57 eV. The peak at 285.25 eV is allocated to C– C/C–H^35^ and is recognized to APTES. A smaller peak at 286.8 is associated with a carbon bound to nitrogen (C–N) of amine (–CH_2_–NH_2_).^36^ The peak at 289.57 eV is assigned to the C–Si bond in APTES/ZnO QDs. These key peaks for APTES in APTES/ZnO QDs are ascribed to the strong capping formation.

The deconvoluted profile of the O 1s peak in ZnO QDs, Fig. 3(d), has two Gaussian peaks at 531.22 and 532.76 eV, while that in the case of APTES/ZnO QDs displays three components at 530.65, 532.01 and 532.78 eV. These peaks are related to low (LP), middle (MP) and high (HP) binding energy peaks, respectively. Sahai *et al.*^37^ assigned these peaks to O^2−^ ions at the intrinsical sites, O^2−^ ions in the oxygen-deficient region and chemisorbed oxygen, respectively. The HP is attributed to the chemisorption and dissociated oxygen or hydroxyl species on the surface of ZnO QDs.^38^ However, Wang *et al.*^39^ attributed the LP to the Zn–O bond rather than the oxygen state. Accordingly, the two Gaussian peaks centered at 531.22 and 532.76 eV in the case of ZnO QDs are attributed to MP and HP, respectively. The deconvoluted profiles of the oxygen peak of APTES/ZnO QDs, in this figure, consisted of three components at 530.65, 532.01 and 532.78 eV, which are attributed to MP, O–Si and HP, respectively.^40^Fig. 3(e), presents N 1s peaks recorded one peak at 400.6 eV (100%) and confirms the neutral amine nitrogen (N–H) of NH_2_ group bounded to C–NH_2_ in APTES.^41,42^ Also, Fig. 3(f) shows the Si 2p core-level spectrum with a main peak at 101.54 eV (100%) and is assigned to Si atom bounded to oxygen and carbon (C–Si–O) in the APTES molecule.^43,44^ These results confirm that the successful capping APTES is grafted or capped with ZnO QDs and is consistent with the FTIR and EDX analysis.

### 3.2. Crystallinity studies

**Fig. 4:**
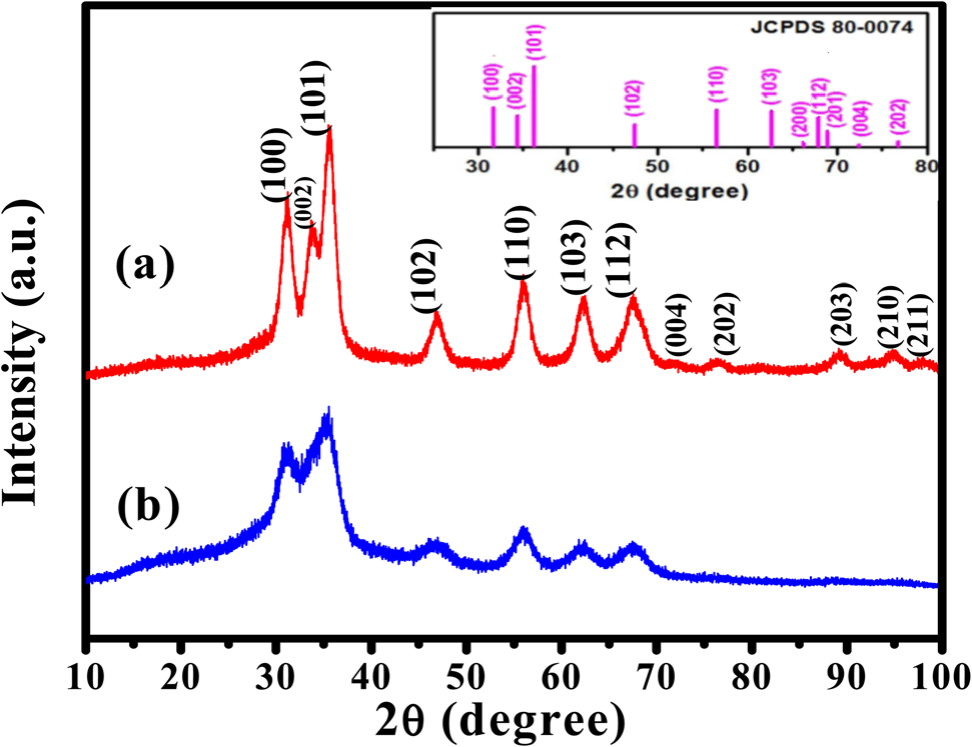
XRD patterns of (a) ZnO QDs and (b) APTES/ZnO QDs. The photograph is standard JCPDS pattern for ZnO QDs.

The crystallinity of the prepared ZnO QDs and APTES/ZnO QDs samples are inspected utilizing XRD analysis and shown in Fig. 4. The shape and peak intensity of the major diffraction peaks presented between 10 and 100° illustrate the high crystallinity of the products. The characteristic peak intensity of APTES/ZnO QDs is lower than that of uncapped ZnO QDs, and the other peaks are disappeared. The pattern of APTES/ZnO QDs has more broad diffraction peaks than uncapped ZnO QDs. These properties are referred to as the coating of ZnO QDs with APTES producing a weak diffraction peak, and the other peaks also disappear. The diffraction peaks at 31.84, 34.63, 36.37, 47.75, 56.74, 62.87, 67.96, 72.57, 76.98, 89.64, 93.08 and 95.63° are corresponded to the (100), (002), (101), (102), (110), (103), (112), (004), (202), (203), (210) and (211) planes, respectively. These data are consistent with the card and typical hexagonal wurtzite-type ZnO crystal structure diffraction pattern (JCPDS card no. 80-0074).^45^ It is noted that the nearby peaks overlap, such as the overlapping of (110), (103) and (112), as a result of the widened diffraction peaks, which illustrates that the QDs have a small grain size.^45^ The diffraction peaks can be indexed to a hexagonal structure with unit cell constants of *a* = 0.324 nm and *c* = 0.518 nm. Compared with the standard diffraction patterns, the prepared ZnO QDs and APTES/ZnO QDs are pure QDs. The combination of the structure of both APTES and ZnO changes the obtained peak's intensity, and some peaks disappear due to the effect of the capping agent. The properties of polycrystalline materials depend on the crystallite size. Debye–Scherrer's eqn (4)^46^ that correlates the peak broadening with the quantum dots size to calculate the crystalline particle size (*D*) is:4
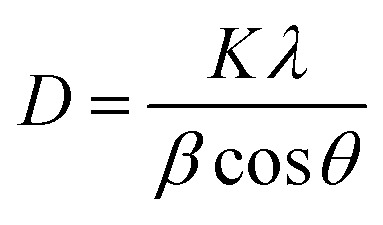
where *β* is the diffraction peak's full width at half-maximum (FWHM), *K* is a Scherrer's constant, which equals 0.89, *λ* is the X-ray wavelength (1.5406 Å), and *θ* is the Bragg diffraction angle. The calculated capped and uncapped ZnO QD's average sizes are evaluated to be 2.04 and 3.64 nm, respectively, consistent with HRTEM results confirming the formation of QDs.

### 3.3. Morphological properties

**Fig. 5:**
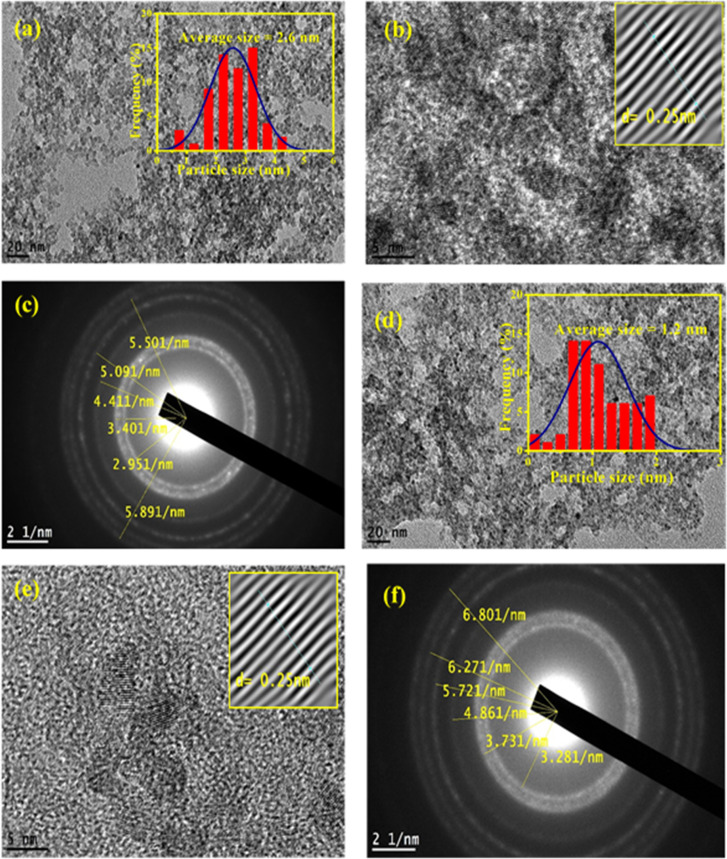
HRTEM images of (a) ZnO QDs. The inset is the particle size distribution of ZnO QDs, (b) ZnO QDs at higher magnification. Inset is *d* spacing of ZnO QDs, (c) SAED of ZnO QDs, (d) APTES/ZnO QDs. The inset is the particle size distribution of APTES/ZnO QDs, (e) high magnified APTES/ZnO QDs. Inset is *d* spacing of APTES/ZnO QDs and (f) SAED of APTES/ZnO QDs.

The HRTEM images of ZnO QDs and APTES/ZnO QDs are revealed in Fig. 5. Fig. 5(a) and (d) indicates that both QDs are spherical. The images in Fig. 5(b) and (e) and the inset in these images also show ZnO QDs and APTES/ZnO QDs at high magnification, respectively. The lattice fringes with *a* spacing of 0.25 nm agree with the inter-planar distance of (002) plane of the hexagonal wurtzite ZnO and confirm the formation of ZnO QDs.^24^ The inset histograms, Fig. 5(a) and (d), present the average size distribution of ZnO QDs and APTES/ZnO QDs with mean diameters of 2.6 nm and 1.2 nm, respectively. The smaller size of APTES/ZnO QDs than ZnO QDs is attributed to inhibiting the crystal growth of the ZnO core in the existence of APTES as a capping agent. The fringes of SAED patterns of ZnO QDs and APTES/ZnO QDs depicted in Fig. 5(c) and (f), are corresponded to different crystal planes of ZnO. The patterns indicate that ZnO QDs are more crystalline than the capped one. The decrease in crystallinity of the APTES/ZnO QDs to uncapped ZnO QDs might be due to the silane coating surface of ZnO QDs not observed in these images.

### 3.4. Surface charges and optical properties

**Fig. 6:**
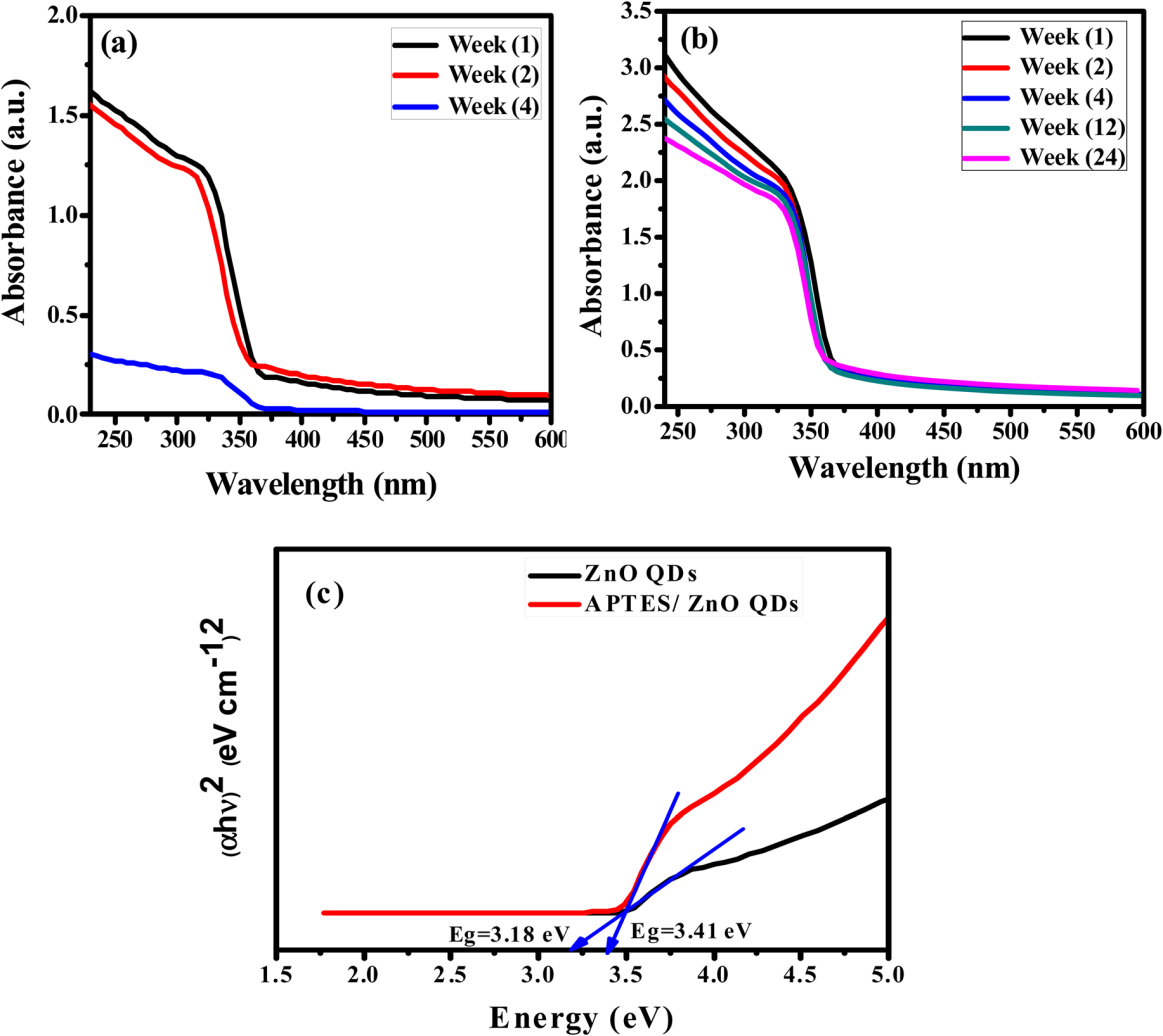
UV-vis spectra of (a) ZnO QDs and (b) APTES/ZnO QDs *vs.* time. (c) Band gap energy of ZnO QDs and APTES/ZnO QDs.

The absorbing characteristics for the prepared ZnO QDs and APTES/ZnO QDs are studied utilizing UV-visible absorption spectroscopy. The spectra of ZnO QDs and APTES/ZnO QDs absorption are recorded in the *λ* range of 250–600 nm and are revealed in Fig. 6(a) and (b). The spectra of both species display only a shoulder at 330 nm. Zhao *et al.* and others reported that ZnO QDs exhibit absorption peaks from 250 to 400 nm.^7,47,48^ Therefore, the shoulder position can be taken as evidence that the ZnO QDs are formed and assigned to the intrinsic band-gap of Zn–O absorption.^47^ The high-intensity shoulder of the APTES/ZnO QDs can be referred to APTES coated surface of ZnO QDs from surface defects and is related to the particle size effect. The stability of ZnO QDs and APTES/ZnO QDs is determined by the intensity of their absorption peaks over time. As presented in Fig. 6(a), the peak intensity of ZnO QDs highly declined by approximately 81% after 4 weeks with a red shift to 340 nm. This is attributed to the coagulation of ZnO particles. On the other hand, APTES/ZnO QDs retain about 94% of their absorbance intensity, and this indicates that APTES/ZnO QDs have high stability without peak shift owing to the quantum confinement effect as shown in Fig. 6(b). Additionally, the colloidal solution of APTES/ZnO QDs maintains its homogeneity and dispersion without any aggregation or alternation of color. It can conclude that APTES act as a stabilizing, capping agent and protects the ZnO QDs from photo-degradation.

The apparent optical band gap energies (*E*_g_) of the prepared capped and uncapped ZnO QDs can be estimated utilizing Tauc eqn (5) through the absorption spectra:^49^

5 *αhν* = *B*(*hν* − *E*_g_)^*n*^

where *α* is the absorption coefficient, *hν* is the photon's energy, *B* is an effective masses constant related to the valence band and conduction band, and *n* is a number that describes the nature of the electronic transition between the valence and conduction bands. Its values of 1/2, 2, 3/2, and 3 represent the allowed direct, allowed indirect, forbidden direct, and forbidden indirect transitions, respectively.

In the case of ZnO QDs (allowed direct transition), the value of *n* = ½.^49^ The band gap is estimated using the Tauc plot as presented in Fig. 6(c). This plot gives *E*_g_ by extrapolating the plot of (*αhν*)^2^*versus hν* onto the *x*-axis. The corresponding band gaps obtained are 3.18 and 3.43 eV for ZnO and APTES/ZnO QDs, respectively. The larger value of the *E*_g_ of APTES/ZnO QDs than that of ZnO QDs indicates that the former has a smaller particle size,^50^ which agrees with the outcomes obtained from HRTEM and XRD. The decrease in band-gap of uncapped ZnO QDs is attributable to hydroxyl moiety (–OH) that has been surface-adsorbed around the hexagonal form of the prepared ZnO QD, which may lead to the formation of trap sites, and this result in the reduction of the band-gap energy.^51^

**Fig. 7:**
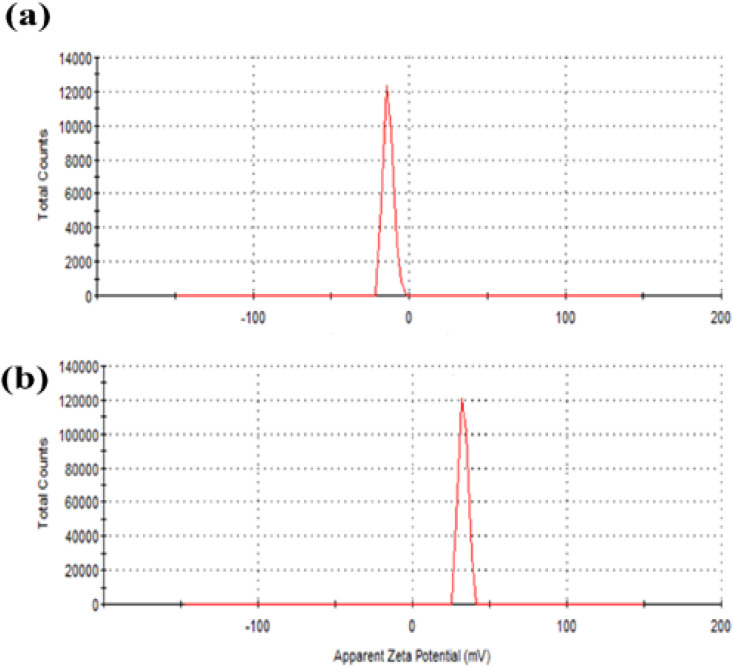
Zeta potential of (a) ZnO QDs and (b) APTES/ZnO QDs.

The efficient electric charge on the QDs surface is measured by zeta potential. Particles with a greater electrostatic repulsion between them show a higher *ξ*-potential with high stability. To further monitor the stability of APTES/ZnO QDs and ZnO QDs, zeta potential (charge of the surface) measurement is performed and shown in Fig. 7(a) and (b). The value of −13.0 mV of zeta potential for ZnO QDs (Fig. 7(a)) indicates that its surface is negatively charged. In contrast, the *ξ*-potential of APTES/ZnO QDs is +33.2 mV (Fig. 7(b)). This large positive charge indicates that APTES stabilizes ZnO QDs. The shift of the *ξ*-potential from −13.0 mV for ZnO QDs to +33.0 mV for APTES/ZnO QDs confirms that APTES is aggregated around the negatively charged ZnO QDs. It has been reported that the potential values greater than +25 mV or lesser than −25 mV represents stable QDs with a low possibility of aggregation.^52^ Accordingly, the prepared APTES/ZnO QDs are stable and not agglomerated.

**Fig. 8:**
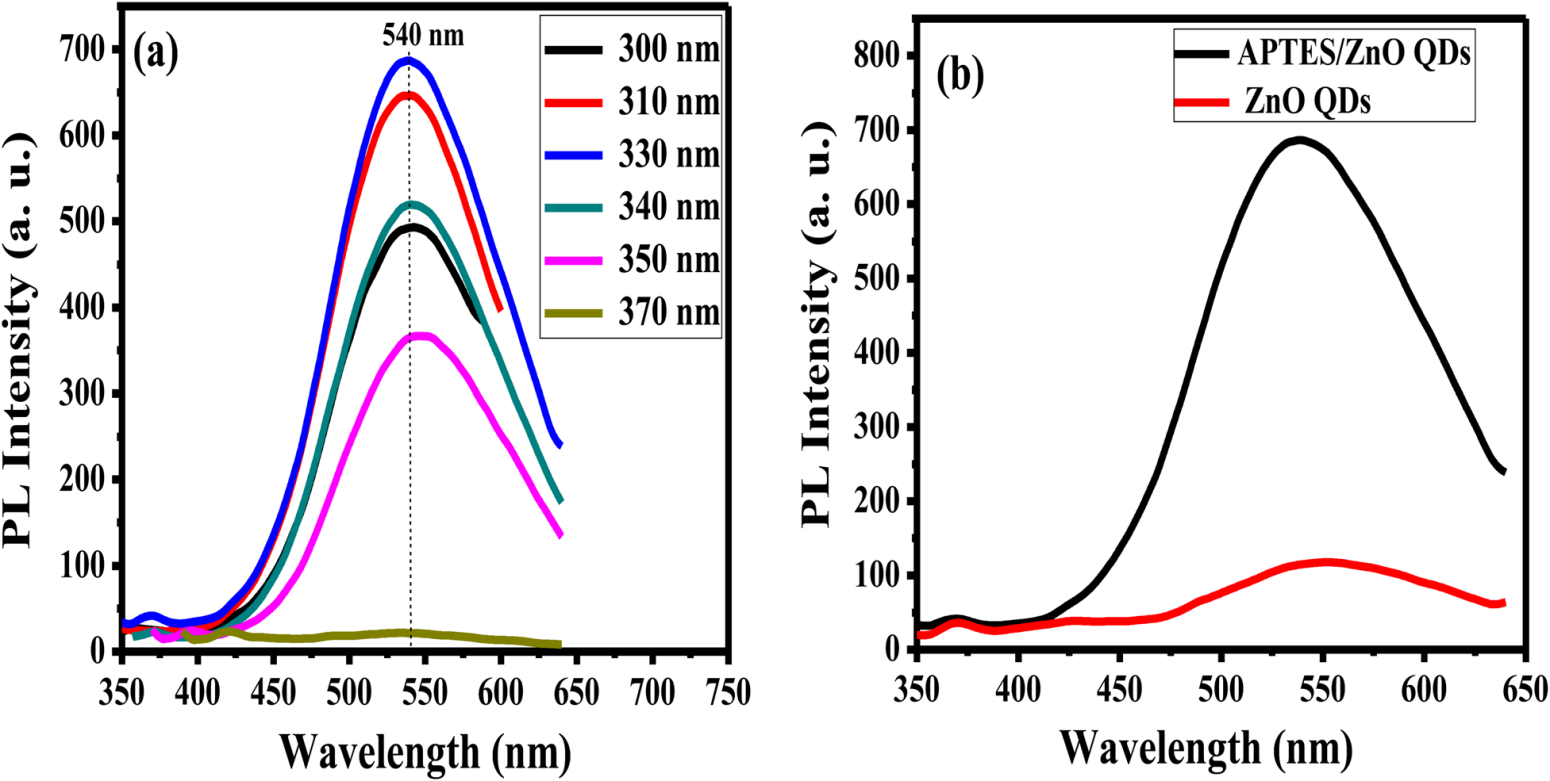
PL spectra of (a) APTES/ZnO QDs at different excitation wavelengths, and (b) APTES/ZnO QDs and ZnO QDs at *λ*_ex_ = 330 nm.

The emission characteristics of APTES/ZnO QDs are examined *via* PL intensity measurements of these QDs at several excitation wavelengths (*λ*_ex_) ranging from 300 nm to 370 nm as revealed in Fig. 8(a). APTES/ZnO QDs exhibit almost *λ*_ex_ independent behavior at *λ*_em_ of 540 nm that can be explained by the narrow size distribution of the QDs or the presence of a specific deep level connected to a defect in ZnO QDs.^53,54^ The PL intensity of APTES/ZnO QDs is raised by increasing the *λ*_ex_ from 300 nm, and a maximum PL intensity is attained at *λ*_ex_ of 330 nm. Furthermore, *λ*_ex_ over 330 nm leads to a decline in PL intensity. When the photons are energetic enough to excite the electrons from the valence band to the conduction band, the PL intensity is enhanced till reach its maximum, which resembles the band-gap energy absorbed by APTES/ZnO QDs. Contrastingly, the advancement of electrons from the valence band to the conduction band occurs when *λ*_ex_ surpasses the band gap. When a second photon with a longer wavelength is emitted, the excited electrons return to their ground state.^54,55^

PL spectrum of uncapped ZnO QDs at *λ*_ex_ of 330 nm has a broader emission peak at *λ*_em_ 550 nm than the *λ*_em_ of APTES/ZnO QDs (540 nm), as displayed in Fig. 8(b). However, the strongest of the luminescent intensity of APTES/ZnO QDs indicated that the surface defects and traps of ZnO QDs are greater than that of APTES/ZnO QDs. The existence of APTES capped on the ZnO QDs lattice surface act as a charge electrons trapping and creates a barrier to keep the electron inside the ZnO QDs internal shell. Subsequently, APTES/ZnO QDs enhance emission.^56^ The red PL shift of uncapped ZnO QDs confirms that the lower band-gap resulting from, the larger QDs size produces broad and shift higher emission (red shift).^57^

**Fig. 9:**
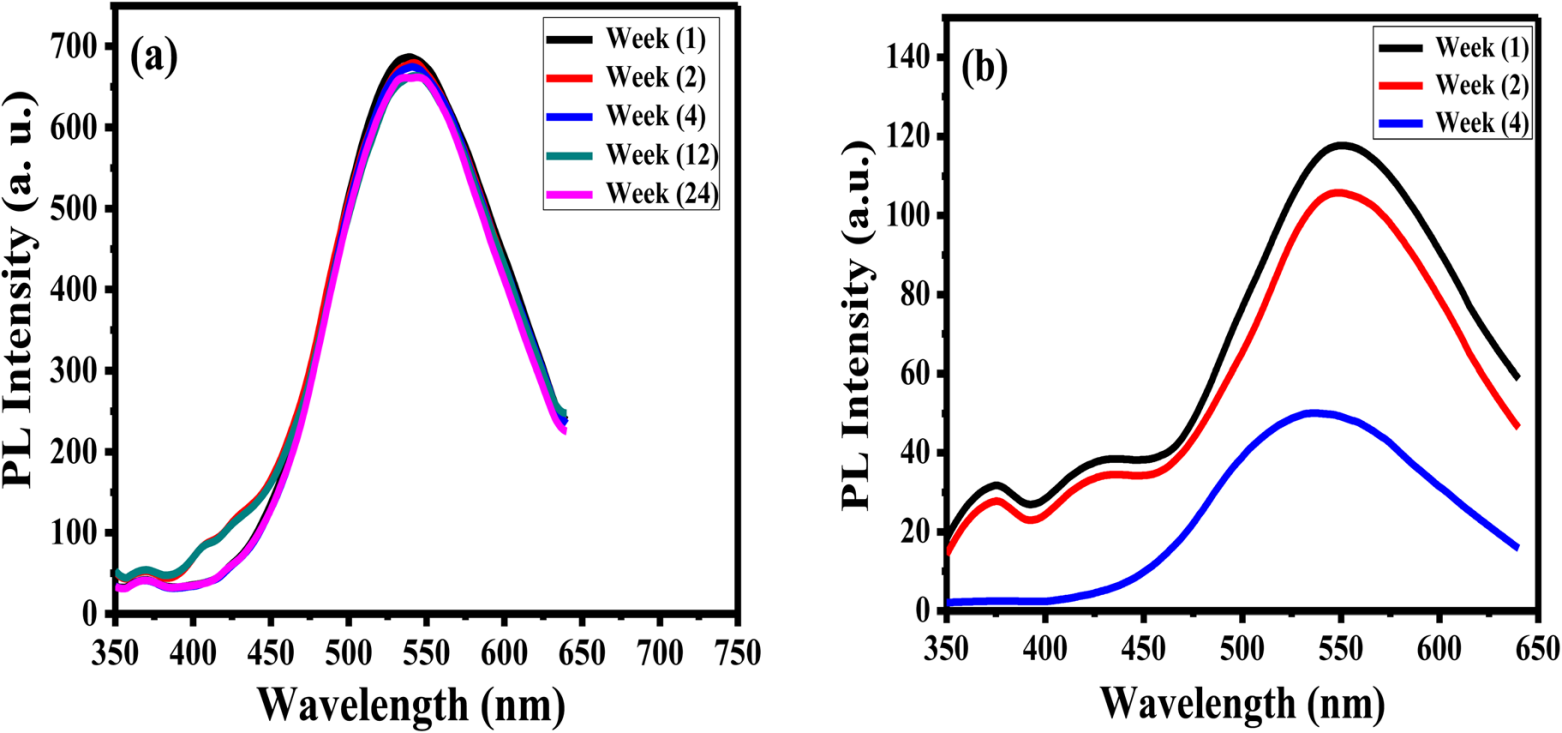
PL spectra of (a) APTES/ZnO QDs and (b) ZnO QDs *vs.* time at *λ*_ex_ = 330 nm.

The stability of luminance of APTES/ZnO QDs and ZnO QDs is investigated by measuring and comparing their PL intensities at *λ*_ex_ 330 nm at different times at room temperature as presented in Fig. 9. It is observed that after six months (24 weeks), the PL intensity of APTES/ZnO QDs remains almost the same and acquires an excellent PL stability over time (Fig. 9(a)). In contrast, PL intensity of uncapped ZnO QDs (Fig. 9(b)) is reduced after two weeks and highly dropped after four weeks with a peak position shift from 540 nm to 530 nm. This suggests that the PL intensity of APTES/ZnO QDs is highly stable over a long time and is a better candidate than uncapped ZnO QDs for sensing applications.

**Fig. 10:**
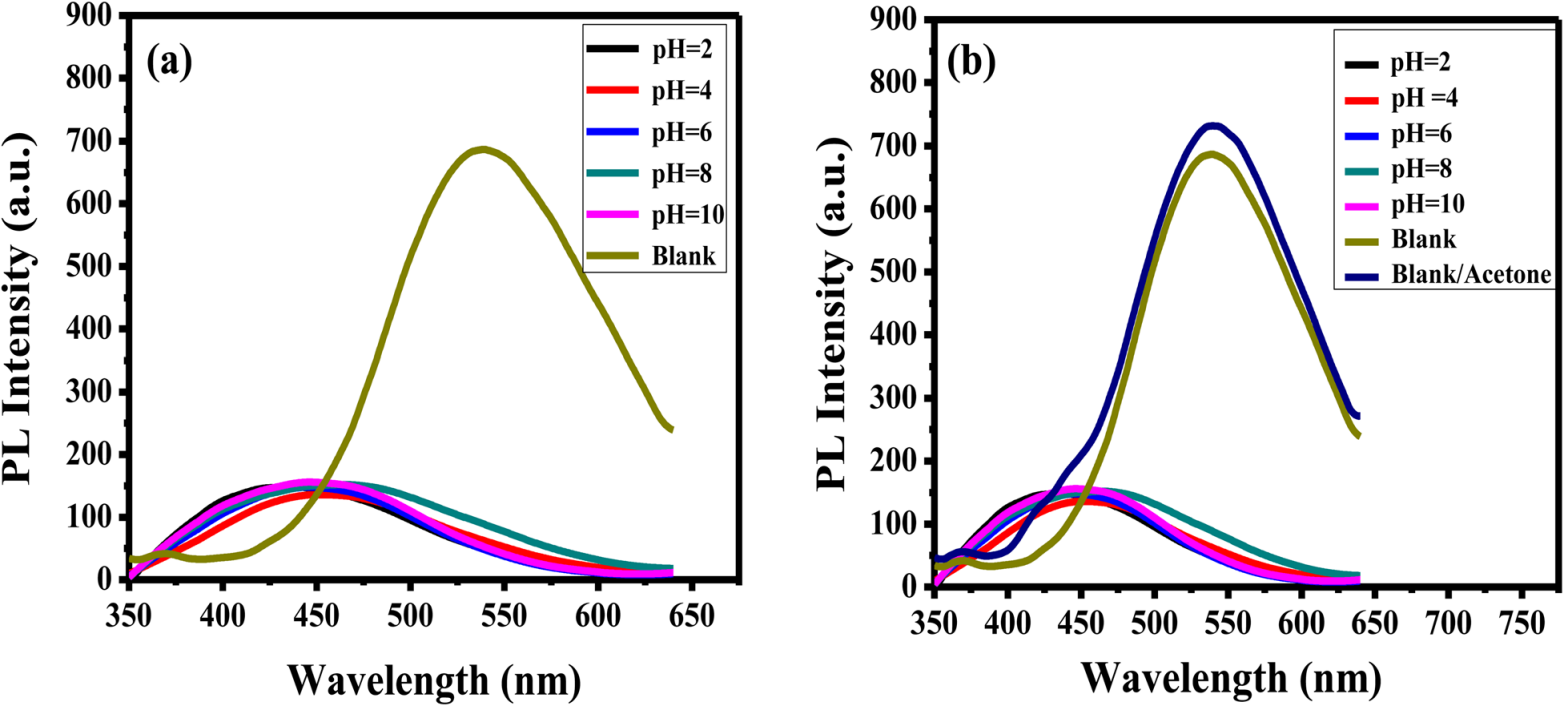
PL spectra of APTES/ZnO QDs (a) in the absence and (b) in the presence of 1.0 mM acetone solution at different pHs at *λ*_ex_ = 330 nm. Blank: APTES/ZnO QDs in the absence of acetone.

**Scheme 1:**
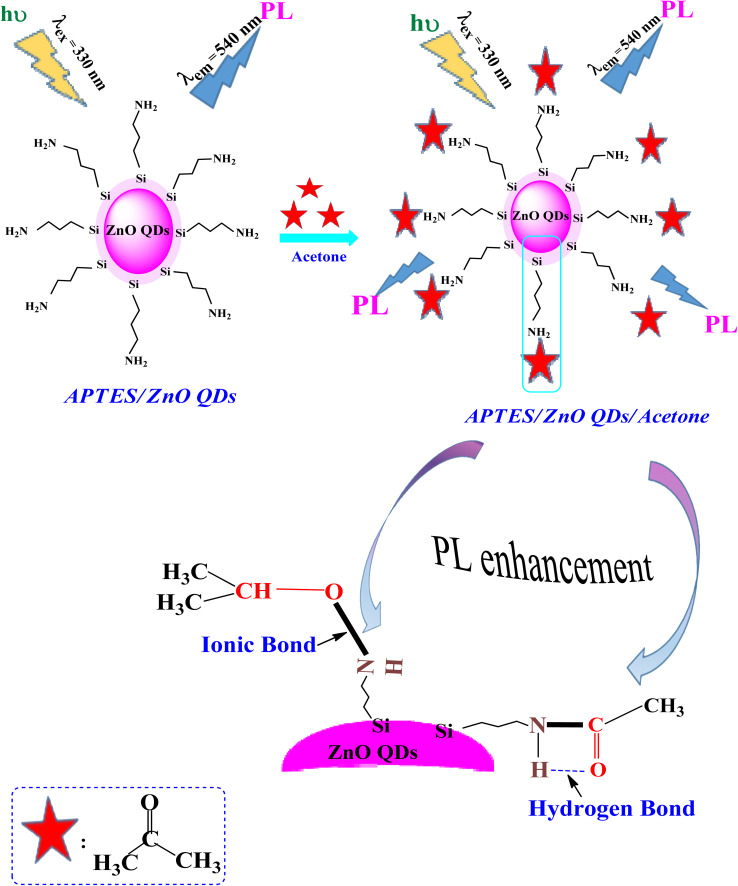
Schematic illustration the interaction between APTES/ZnO QDs and acetone.

The pH of the solution influences accurate analytes detection. To explore the impact of pH on the luminance of the APTES/ZnO QDs on the acetone detection, the PL emission of APTES/ZnO QDs is examined and carried out in the absence (blank), and the presence of 1.0 mM acetone in pH ranging from 2 to 10 as revealed in Fig. 10(a) and (b). APTES/ZnO QDs (0.1 mL) were added to 9.9 mL of different pHs of PBS. Fig. 10(a), reveals that PL spectra of APTES/ZnO QDs at different pHs. The emissions appeared at 450 nm compared with the blank of APTES/ZnO QDs in ethanol with a pH of 8.9 at 540 nm. It is also observed that blank is presented the highest PL intensity. Fig. 10(b) displayed the influence of pH on the PL efficiency and enhancement of APTES/ZnO QDs in the presence of 1.0 mM acetone. It is observed that the PL dependence on the pH has the same trend in the acetone absence. It is noticed that the addition of acetone increased the PL intensity of the blank sample, while there was no effect on the other samples with different pHs. The PL enhancement will be explained in detail in the sensing mechanism (Scheme 1). This PL response is attributed to the fact that PBS affect the functional groups of APTES/ZnO QDs such as amine groups (NH_2_) and cause protonation or deprotonation of these functional groups in acidic or basic media.^8,58^ It can be concluded that blank is selected for further analytical experiments.

### 3.5. Performance of APTES/ZnO QDs for acetone sensing

**Fig. 11:**
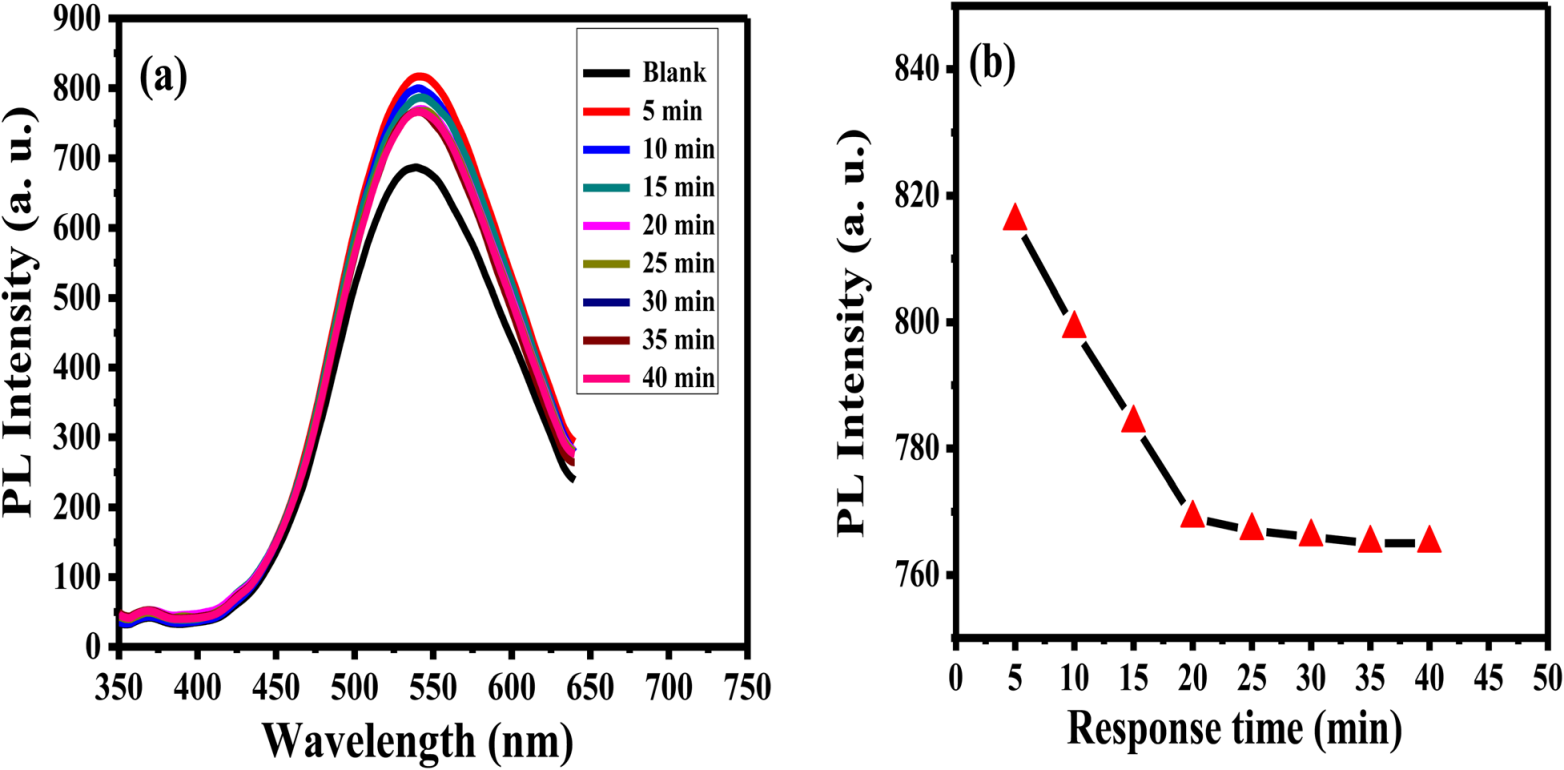
PL spectra (a) and PL intensity (b) of APTES/ZnO QDs in presence of 1.0 mM acetone *versus* response time at *λ*_ex_ = 330 nm.

The enthusiastic photo-luminance of APTES/ZnO QDs is a crucial feature for its use as an acetone sensor. The influence of the response time of 1.0 mM acetone with APTES/ZnO QDs on PL intensity is studied as presented in Fig. 11(a) and (b). The PL intensity of APTES/ZnO QDs is enhanced quickly after adding the acetone in the first 5 min; then, it slightly declined to 25 min. After 25 min, PL intensity is attained to the plateau region. This is explained by the transition among the valence and conduction bands dependent on band edge broadening or enlargement. In addition, the effective mass difference between electrons in the conduction band and holes in the valence band initiates this declining trend.^59^ These results revealed that APTES/ZnO QDs are reactive and stable toward acetone. Therefore, 25 min is the optimal working time for detecting acetone.

**Fig. 12:**
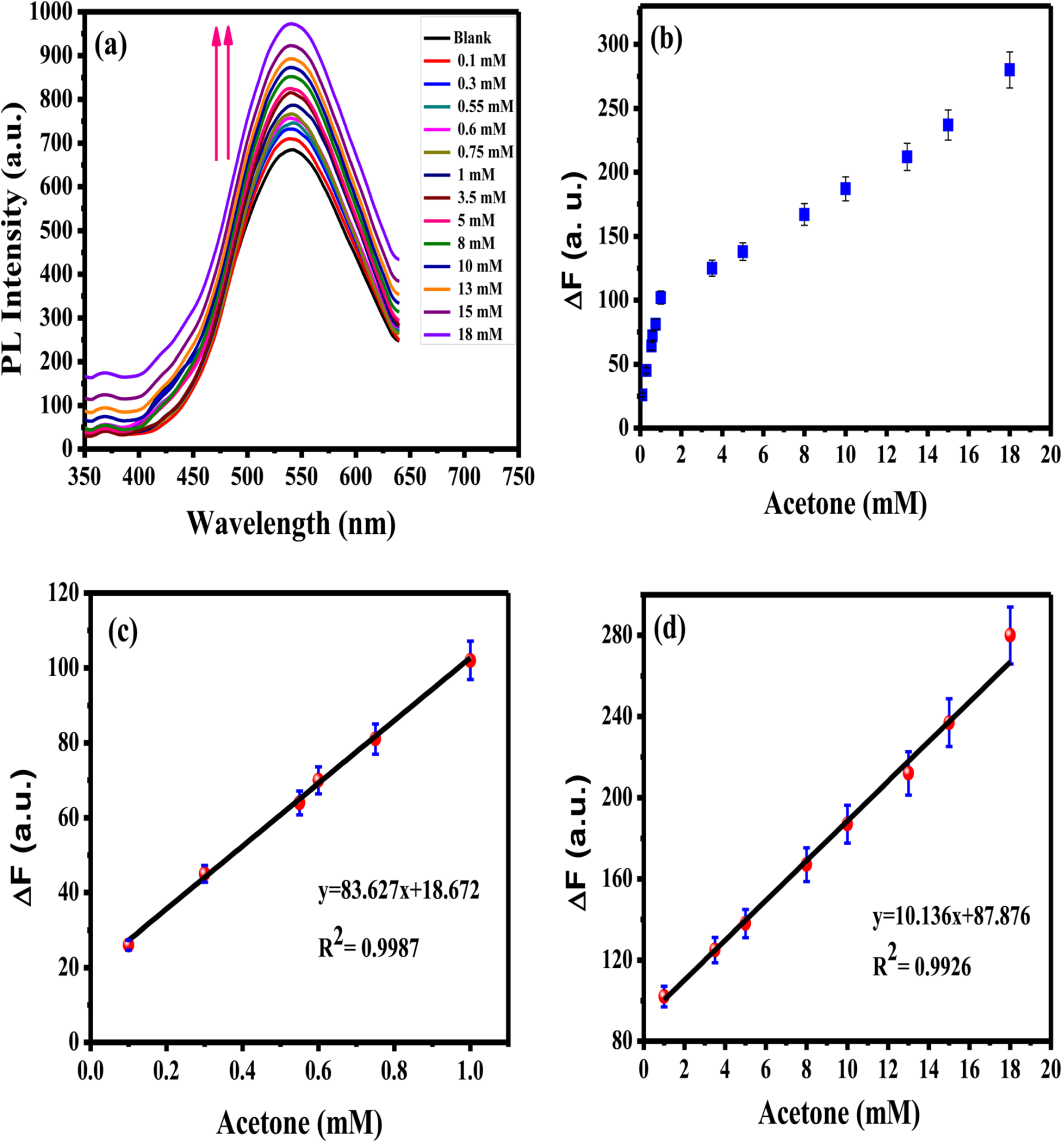
(a) PL spectra of APTES/ZnO QDs with various acetone concentrations, and Δ*F vs.* acetone concentration from (b) 0.1 to 18 mM, (c) 0.1 to 1 mM, (d) 1 to 18 mM at *λ*_ex_ = 330 nm.

Sensitivity, linearity, dynamic range, and detection limit of APTES/ZnO QDs probe in the existence of acetone concentrations sequences ranging from 0.1 to 18.0 mM are determined using PL property as displayed in Fig. 12(a)–(d). It is observed that the rising acetone concentration from 0.1 to 18.0 mM provokes an enhancement of the luminescence, as shown in Fig. 12(a). This enhancement in the PL intensity might be due to the addition of acetone to the QD solution that diminished the surface QDs defects, and this will be explained in the interaction mechanism (Scheme 1). Fig. 12(b)–(d) reveals that acetone has a good affinity for APTES/ZnO QDs which show a two linear relationship between the PL enhancing intensity (Δ*F*)^22^ and acetone concentration in the ranges of 0.1–1 mM (*R*^2^ = 0.9987) and 1–18 mM (*R*^2^ = 0.9926). This proves that the acetone can be detected quantitatively utilizing APTES/ZnO QDs probe with various sensitivities (the calibration line's slopes) of 83.627 and 10.136 Mm^−1^ can be attained from the detection range. From the calibration curve (Fig. 12(c)), the LOD was calculated to be 0.0424 mM (∼42 μM). It is found that APTES/ZnO QDs have an acceptable limit for acetone detection that is lesser than the permissible limit of 5.0 mM acetone in diabetic urine^23^ and below the allowable of 2.9 mM acetone by WHO.^17^ Therefore, APTES/ZnO QDs luminescent sensor is sensitive enough to detect acetone concentration in an actual sample.

### 3.6. Interaction mechanism between APTES/ZnO QDs and acetone

Scheme 1 clarifies the PL enhancement of APTES/ZnO QDs for acetone detection through two mechanisms. APTES is covalently bonded to anchored on the ZnO QDs surface through silica to obtain the APTES/ZnO QDs. The literature showed that compounds containing the NH_2_ group enhance the PL intensity.^60^ The main mechanism involves strong emission associated with the conjugation between amino groups (electron donor) of the APTES/ZnO QD surface and acetone carbonyl groups through NH–CO linkage, providing electrons to bind with acetone and hydrogen bond formation, and leads to PL enhancement.^61^ Otherwise, when acetone is absorbed by APTES/ZnO QDs surface, it may be formed ionic bond between nitrogen cation of APTES and oxygen anion of acetone.^62^ Subsequently, acetone is chelated and selected onto the APTES/ZnO QDs surface as the sensing moiety.

The performance of APTES/ZnO QDs as PL sensor for acetone is compared to other techniques reported in previous literature as shown in Table 1.

**Table 1:** Comparison of the present method with the published methods for acetone detection

Methods	Detection range	LOD	Ref.
Headspace/gas chromatography	0.2–1.95 mg L^−1^	1.12 mg L^−1^	15
Solid phase single reagent	5–200 mg dL^−1^	5 mg dL^−1^	19
Point of care screen tests	3–30 mM	0.84 mM	23
Ion mobility spectroscopy	5–80 mg L^−1^	5.30 mg L^−1^	63
High performance liquid chromatography (HPLC)	0.5–20 mM	0.136 mM	64
Resistive conducting polymer sensor	0.005–25 mM	8.3 mM	65
Fluorescence	0.5–150 mM	0.5 mM	66
Fluorescence	0.1–18 mM	0.0424 mM	This work

### 3.7. Reproducibility and repeatability studies

**Fig. 13:**
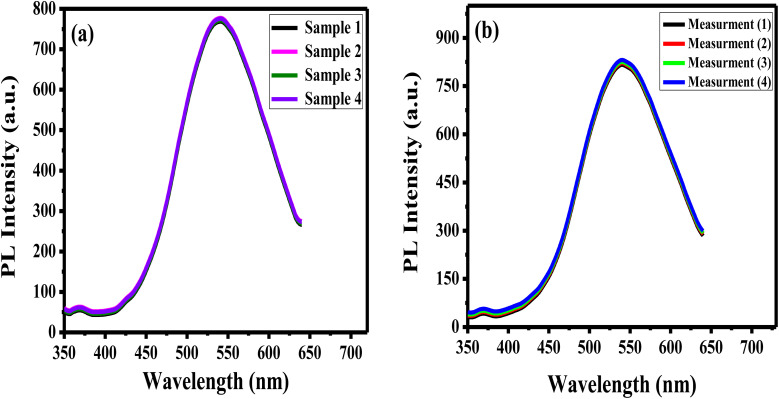
(a) The reproducibility of APTES/ZnO QDs for PL measurements of 4 different samples containing 0.8 mM acetone, and (b) repeatability of APTES/ZnO QDs for 4 independent PL measurements of a sample containing 4 mM acetone.

The reproducibility of the prepared APTES/ZnO QDs sensor is estimated when measuring the PL intensity of four different samples of APTES/ZnO QDs with acetone (0.8 mM) solutions prepared separately (Fig. 13(a)) and the RSD is found to be 2.2%. Additionally, the synthesized sensor's repeatability is verified after 4 consecutive PL measurements of the same APTES/ZnO QDs with 4 mM acetone solution as depicted in Fig. 13(b). The RSD is found to be 2.4%. These results confirm that the APTES/ZnO QDs probe is extremely reproducible and suitable for repetitive measurements.

### 3.8. Selectivity and interferences of APTES/ZnO QDs

**Fig. 14:**
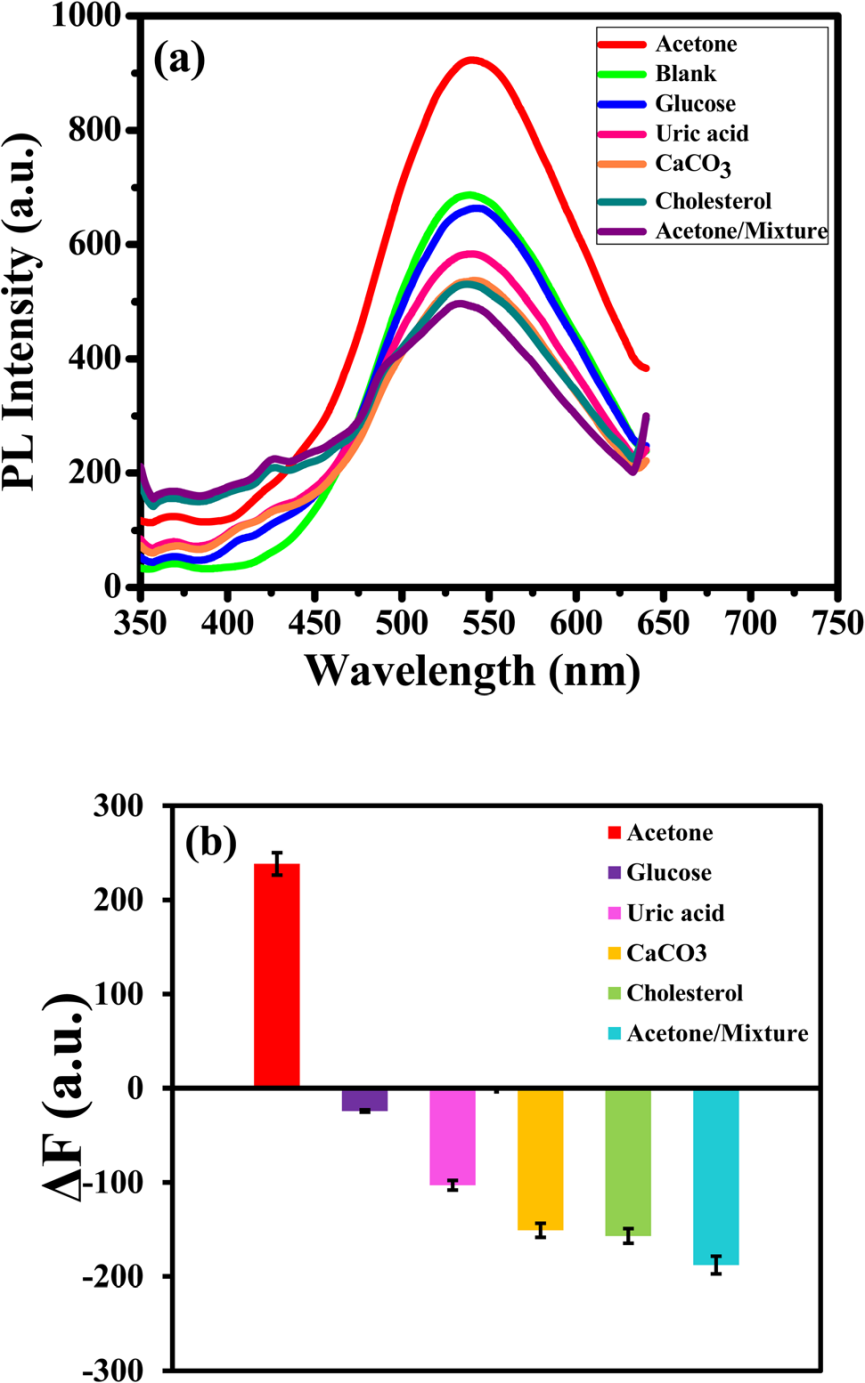
(a) PL spectra and (b) Δ*F* of APTES/ZnO QDs for different bioanalytics at *λ*_ex_ = 330 nm.

Selectivity is a crucial operator in assessing the effectiveness of the luminous sensor. The selectivity of the assay is examined with different bioanalytics presented in human urine and interfered such as glucose, uric acid, calcium carbonate and cholesterol and their mixture. The PL response and the PL enhancing intensity (Δ*F*)^22^ change of APTES/ZnO QDs at a concentration of 15.0 mM of various bioanalytics is recorded as shown in Fig. 14(a) and (b). It is found that glucose, uric acid, calcium carbonate and cholesterol have a quenching impact on the PL intensity of APTES/ZnO QDs. Otherwise, acetone is the only bioanalytics with a strong enhanced PL response, clarified through columns in Fig. 14(b). The anti-interference studied when all co-existing analytes did not induce appreciable change in the fluorescence emission of sensor.^67^ These results confirm the superior selectivity of APTES/ZnO QDs as a biosensor probe toward acetone. They can be practically applied in an actual urine samples.

### 3.9. Sensing of acetone in an actual human urine sample

**Fig. 15:**
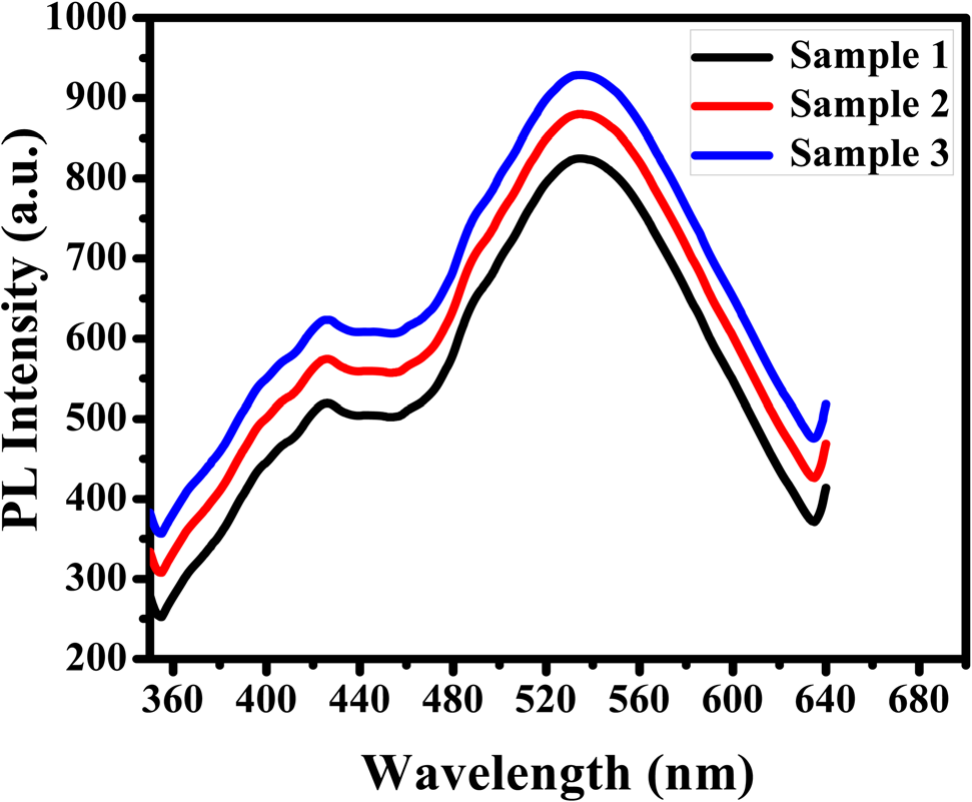
PL spectra of APTES/ZnO QDs with diabetic urine samples at *λ*_ex_ = 330 nm.

The efficiency of APTES/ZnO QDs as a biosensor of acetone in human urine samples is tested. Three urine specimens from diabetic patients are obtained from a medical laboratory with their clinical acetone concentrations. Then, the PL spectrum of the urine sample is measured individually (Fig. 15), and acetone concentration is detected *via* the previous calibration curve (Fig. 12(d)). This experiment is repeated in triplicate for each urine sample to improve reliability. These results are recorded in Table 2, illustrating that the acetone level is 4.85–15.11 mM, corresponding to the recovery percentages calculated and found in the 97–102.7% range. The RSD is estimated to be 2.5, 2.6 and 2.5% for samples (1), (2) and (3), respectively.

**Table 2:** The measured and recovered acetone concentration in a human diabetic urine samples using APTES/ZnO QDs probe^a^

Sample	Clinical data provided (mM)	Acetone found (mM)	Recovery (%)	RSD (%)
(1)	5.00	4.85	97	2.5
(2)	10.00	10.27	102.7	2.6
(3)	15.00	15.11	100.7	2.5

a Recovery% = (concentration found/concentration measured) × 100%.

These results confirmed that the prepared APTES/ZnO QDs are successfully applied to detect acetone levels accurately and efficiently and can be used in actual biological fluids.

## 4 Conclusion

In this paper, the successful preparation of both ZnO QDs and APTES/ZnO QDs was achieved and confirmed by using several characterization techniques. The APTES/ZnO QDs biosensor offered an extended time for the detection application. APTES/ZnO QDs were used as a sensitive fluorescence probe for acetone detection in the wide range from 0.1 to 18.0 mM providing a good a low LOD of 0.0424 mM. The PL enhancement mechanisms are possibly due to hydrogen bond or ionic bond formation. Furthermore, the APTES/ZnO QDs biosensor offered a high selectivity to acetone in other interfere such as glucose, uric acid, calcium carbonate, and cholesterol. Also, the APTES/ZnO QDs displayed perfect reproducibility and repeatability with RSD of 2.2% and 2.4%, respectively. In addition, the calculated recovery percentages of acetone were found in the range of 97–102.7% which confirmed that APTES/ZnO QDs can be used an effective tool for acetone sensing in biological fluids in the future.

## Author contributions

Goerget Saber: conceptualization, data curation, formal analysis, investigation, methodology, writing – original draft. Gamal Badie: supervision – review & editing. Ali El-Dissouky: conceptualization – supervision – review & editing. Shaker Ebrahim: conceptualization, supervision, data curation, formal analysis, writing – review & editing. Azza Shokry: conceptualization, supervision, data curation, formal analysis, writing – review & editing.

## Conflicts of interest

The authors declare that they have no known competing financial interests or personal relationships that could have appeared to influence the work reported in this paper.

## Informed consent

Informed consent was obtained from all individual participants included in the study.

## Supplementary Material
